# Multicenter Study of Dynamic High-Density Functional Substrate Mapping Improves Identification of Substrate Targets for Ischemic Ventricular Tachycardia Ablation

**DOI:** 10.1016/j.jacep.2020.06.037

**Published:** 2020-12

**Authors:** Neil T. Srinivasan, Jason Garcia, Richard J. Schilling, Syed Ahsan, Girish G. Babu, Richard Ang, Mehul B. Dhinoja, Ross J. Hunter, Martin Lowe, Anthony W. Chow, Pier D. Lambiase

**Affiliations:** a Department of Cardiac Electrophysiology, The Barts Heart Center, St. Bartholomew’s Hospital, London, United Kingdom; b Institute of Cardiovascular Science, University College London, London, United Kingdom; c Royal Bournemouth and Christchurch Hospitals, Bournemouth, United Kingdom

**Keywords:** ablation, functional substrate mapping, late potentials, substrate mapping, ventricular tachycardia, DEEP, decrement-evoked potential, ERP, effective refractory period, LAVA, local abnormal ventricular activation, LP, late potentials, RV, right ventricular, SP, sense protocol, VT, ventricular tachycardia

## Abstract

**Objectives:**

The goal of this study was to evaluate the role of dynamic substrate changes in facilitating conduction delay and re-entry in ventricular tachycardia (VT) circuits.

**Background:**

The presence of dynamic substrate changes facilitate functional block and re-entry in VT but are rarely studied as part of clinical VT mapping.

**Methods:**

Thirty patients (age 67 ± 9 years; 27 male subjects) underwent ablation. Mapping was performed with the Advisor HD Grid multipolar catheter. A bipolar voltage map was obtained during sinus rhythm (SR) and right ventricular sense protocol (SP) single extra pacing. SR and SP maps of late potentials (LP) and local abnormal ventricular activity (LAVA) were made and compared with critical sites for ablation, defined as sites of best entrainment or pace mapping. Ablation was then performed to critical sites, and LP/LAVA identified by the SP.

**Results:**

At a median follow-up of 12 months, 90% of patients were free from antitachycardia pacing (ATP) or implantable cardioverter-defibrillator shocks. SP pacing resulted in a larger area of LP identified for ablation (19.3 mm^2^ vs. 6.4 mm^2^) during SR mapping (p = 0.001), with a sensitivity of 87% and a specificity of 96%, compared with 78% and 65%, respectively, in SR.

**Conclusions:**

LP and LAVA observed during the SP were able to identify regions critical for ablation in VT with a greater accuracy than SR mapping. This may improve substrate characterization in VT ablation. The combination of ablation to critical sites and SP-derived LP/LAVA requires further assessment in a randomized comparator study.

Ventricular tachycardia (VT) is classically associated with re-entrant arrhythmia over a fixed anatomical structure. Activation and entrainment mapping of VT remain the gold standard for identifying critical sites for ablation of VT (1); however, this method is limited by poorly tolerated or nonsustained VT. Several substrate-guided approaches have been developed to overcome this, including scar homogenization (2) and late potentials (LP) mapping (3, 4, 5). However, outcomes when comparing both methods are similar (6), and procedure success can be as low as 47% (7).

A key element in facilitating VT is the presence of dynamic changes within the substrate that may not be evident during sinus rhythm substrate mapping but may form a critical aspect of the tachycardia mechanism when conduction velocity slows dynamically and tissue refractory periods lengthen. We have previously described dynamic substrate changes within regions of myocardial scar and LP (8). Several methods have been studied to invoke dynamic substrate changes in critical regions for ablation, including decrement-evoked potential (DEEP) mapping (9,10), which involves a drive train and S2 pacing protocol, and evoked delayed potential mapping, in which electrogram changes during right ventricular (RV) drive train pacing and S1 to S2 pacing are examined (11). However, VT on device traces is often seen to be initiated by single extrasystolic beats (12,13).

The current study aimed to investigate dynamic substrate changes to local abnormal ventricular activity (LAVA) and LP, in relation to critical sites for VT ablation using high-resolution mapping of the ventricle with the Advisor HD Grid (Abbott, Inc., Abbott Park, Illinois), during short coupled single extrastimuli from the right ventricle (Barts Sense Protocol [SP]), designed to invoke conduction delay. We hypothesized that the dynamic functional substrate mapping would improve the identification of critical substrate.

## Methods

### Patient demographic characteristics

Thirty consecutive patients (mean age 67 ± 9 years; 27 male subjects) with ischemic cardiomyopathy undergoing clinical VT ablation for symptomatic antitachycardia pacing, symptomatic sustained VT, or implantable cardioverter-defibrillator shocks were enrolled from 2 UK centers. Mean ejection fraction was 25 ± 10%. Supplemental Table 1 summarizes the patient cohort. The study conformed to the Declaration of Helsinki, and patients gave informed consent. Research was approved by the local Research Ethics Committee.

### VT mapping and ablation protocol: the barts SP

**Figure 1:**
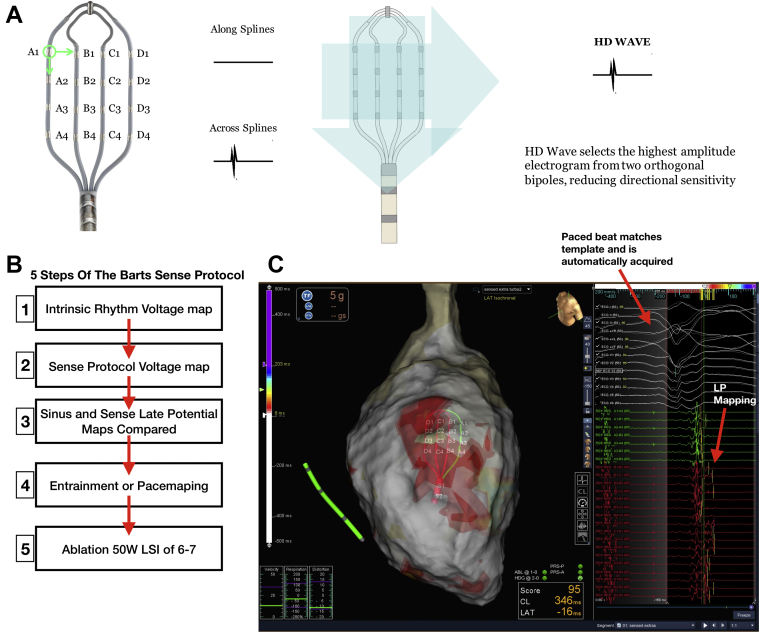
High Density Grid and Sense Protocol Mapping Technique **(A)** High-density (HD) grid and schematic of the HD wave solution. The HD grid consists of 16 equally spaced electrodes arranged in a 4 × 4 grid. Bipolar wave fronts are measured both along and across the splines, with the HD wave solution selecting the highest amplitude signal from 2 orthogonal bipoles, thus obviating the problem of bipolar blindness whereby a wave front traveling across and along the splines would record a low-amplitude signal. **(B)** Schematic showing the 5 steps of the Barts Sense Protocol. Maps of late potentials are made automatically using the TurboMap feature, which allows retrospective maps to be created from the data. **(C)** Example of Barts Sense Protocol mapping for late potentials. The paced beat is templated within the mapping system, and points acquired based on a good score match to the paced morphology. HD grid signal with late potentials can be seen; the latest deflection is demarcated as a yellow line and is created automatically by the mapping system with manual user checks/correction. This creates a timing map based on timing of late potentials post-QRS.

VT Mapping and ablation were performed by using the EnSite Precision mapping system (Abbott, Inc.) (Figure 1A, Central Illustration). Endocardial access to the left ventricle was obtained by using the retrograde arterial or transseptal approaches. A hexapolar catheter was placed in the RV apex for pacing, with the proximal pole located in the inferior vena cava blood pool as the reference for unipolar signals.

**Central Illustration:**
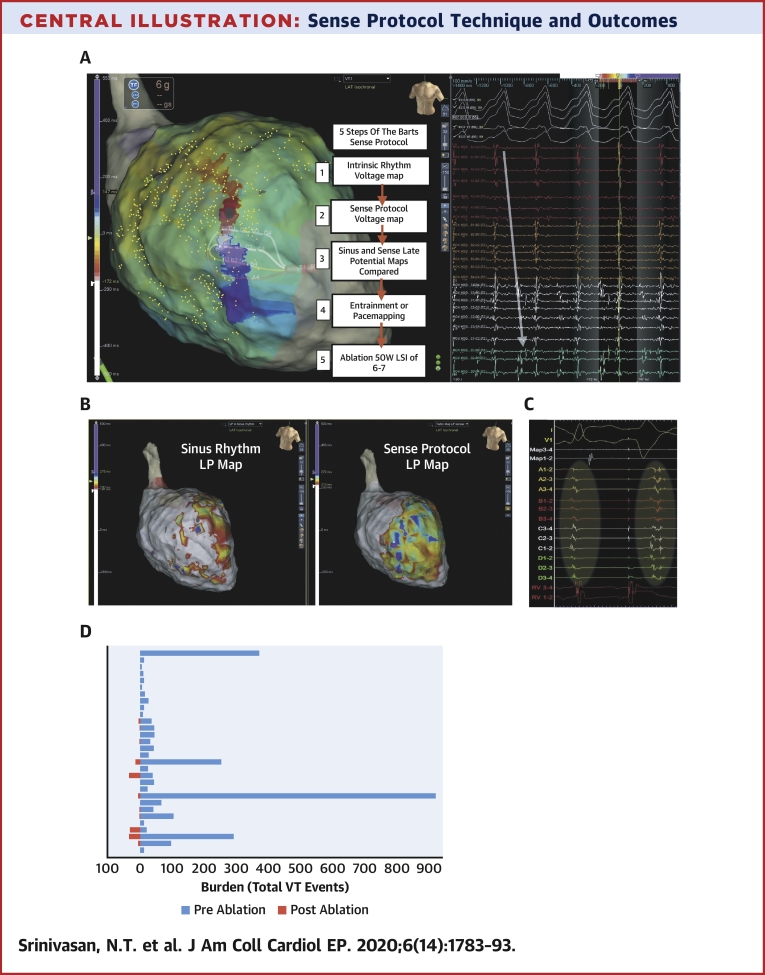
Sense Protocol Technique and Outcomes The Barts Sense Protocol involves creating a substrate map of a single extra packed beat at short a short coupling interval. The 5 steps to the protocol are described in **panel A**. **(A)** Activation mapping of ventricular tachycardia (VT) in a patient with previous anterior myocardial infarction as shown in Video 1. The HD Grid is placed on the critical isthmus, with mid-diastolic signals recorded on the right, it can be seen that the activation sequence goes from spline A to spline D, demarking the entrance and exit of the VT diastolic channel **(white arrow)**. **(B)** Late potential color timing map during sinus rhythm **(left)** and sense protocol pacing **(right)**, showing a greater region of late potentials during the sense protocol corresponding to the mapped the diastolic pathway of VT. **(C)** Example of local late potential delay and splitting of late potentials during sense protocol (second beat) along the mapped diastolic pathway of VT, enabling better characterization of regions of slow conduction critical for ablation. **(D)** Plot demonstrating total VT burden for each patient 6-months pre-ablation and in the follow up period post-ablation.

Two substrate maps were obtained, one during sinus rhythm and one mapping the paced beat of a single sensed extra from the RV apex (Barts SP) to invoke left ventricular conduction delay (Figure 1B). Maps were performed simultaneously by using turbo mapping. The Barts SP involves finding the effective refractory period (ERP) of the single paced RV sensed extra (without a drive train), delivering single sensed extras at 20 ms above RV ERP every fifth beat, templating the morphology of this paced beat, and collecting points that match the template morphology to create a substrate map of this paced beat (Figure 1C). LP were defined, as per published literature (3,5), as isolated high-frequency local electrograms after the offset of the terminal portion of the QRS. To assess local activation time of LP, the window of interest was set at +500 ms from reference.

Following this, activation complemented by entrainment mapping of induced VTs were performed in 21 patients, 9 of whom also had pace mapping performed for additional conformation. The critical isthmus during entrainment mapping was considered according to established criteria (1). Where mapping/entrainment in VT was not possible, a pace map strategy was used (9 of 30 patients); we aimed for a match >96% to the clinical VT, as previously described (14). A total of 75 VTs were entrained (n = 45) or pace mapped (n = 30) in 30 patients. Ablation was then performed to sites of best entrainment/pace map and all LP and LAVA substrates defined by the Barts SP, using the TactiCath Ablation Catheter (Abbott Inc.), irrigated at a power of 50 W, targeting a lesion size index of 7 for each lesion. Procedure endpoint was VT *noninducibility*. *Noninducibility* was confirmed by programmed electrical stimulation, with a drive at 600 ms and 400 ms performed until ERP at S4 from the RV apex and base. Nonclinical VTs were not ablated.

### Data collection and analysis

Substrate maps were collected by using the Advisor HD Grid (Figure 1A), a multipolar mapping catheter containing 16 equally spaced electrodes in a 4 × 4 grid layout. Bipolar voltage maps were collected by using the high-density (HD) wave-mapping technology of the Advisor HD Grid, whereby bipolar recording along and across splines was enabled, with the system analyzing orthogonal bipolar wave fronts and recording the best of the 2 signals to negate the effect of wave front directionality. In addition, the system uses the best duplicate algorithm, whereby the highest amplitude signal in a collected region is displayed on the map. Normal myocardium was defined as tissue with a bipolar voltage >1.5 mV, dense scar was defined as a bipolar voltage <0.5 mV, and scar border zone was defined as a bipolar voltage 0.5 to 1.5 mV, consistent with previously published data (2).

Subsequently, a new window of interest was set within the mapping system that contained the entire diastolic interval, and the TurboMap feature was used to identify the latest LP from the Barts SP data. The system was set to annotate the latest LP identified within the diastolic window, and these were then individually checked and manually corrected (Figure 1C).

### Statistical analysis

Continuous variables are represented as mean ± SD if normally distributed and median (25th to 75th quartile) if not normally distributed. The paired Student’s *t*-test was used to compare differences in location in relation to critical areas for ablation, scar area, and LP between intrinsic rhythm and SP mapping. A p value <0.05 was considered statistically significant. Analysis was performed by using R statistical software (R Foundation for Statistical Computing, Vienna, Austria).

## Results

### Ablation outcomes

Ablation guided by the Barts SP rendered VT noninducible in 29 patients, and 90% (27 of 30) of patients were free from symptomatic VT/antitachycardia pacing or implantable cardioverter-defibrillator shocks at a median follow-up of 12 months. The mean VT burden was reduced from 89 events per patient in the 6 months’ pre-ablation to 4.6 events per patient in the median 12 months’ post-ablation follow-up period; mean shocks per-patient burden decreased from 4.4 to 0.27 in the same time period (Central Illustration, Supplemental Figure 1, Supplemental Tables 2 and 3). Median procedure time was 4 h 6 min, and median ablation time was 32 min.

### Comparison of scar area: intrinsic rhythm versus SP

**Figure 2:**
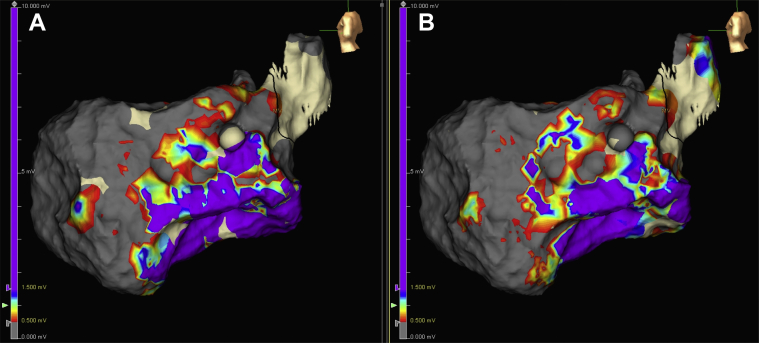
Bipolar Voltage Comparison During Sinus and Sense Protocol Substrate maps during intrinsic rhythm **(A)** and “sense protocol” pacing **(B)** in a patient with previous anterior myocardial infarction requiring ventricular tachycardia ablation. Comparison of bipolar voltage during intrinsic rhythm and sense protocol mapping shows similar delineation of scar region. Bipolar voltage was set to conventional criteria with dense scar <0.5 mV in gray, normal voltage >1.5 mV in purple, and scar border zone 0.5 to 1.5 mV. Color bar indicates voltage to the left of each map.

Figure 2 presents an example of bipolar scar voltage comparison during intrinsic rhythm and during SP mapping in a sample patient. It can be seen that there are no major differences in the scar area when comparing intrinsic rhythm versus the SP while mapping using the HD wave solution and the best duplicate algorithm. The mean dense scar area was 38.2 ± 34 mm^2^ during intrinsic rhythm and 37.4 ± 34 mm^2^ during the SP map, with no statistically significant differences between the two (p = 0.25).

### Comparison of LP and LAVA identification: intrinsic rhythm versus SP

**Figure 3:**
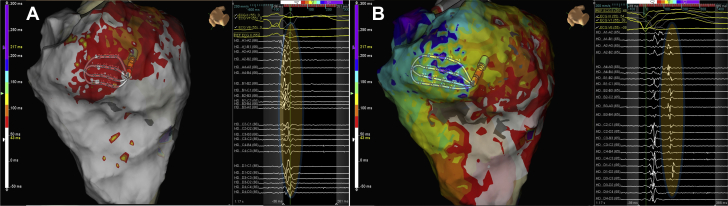
Dynamic Functional Late Potential Unmasking by the Sense Protocol Color maps of late potentials (last deflection) during intrinsic rhythm **(A)** and sense protocol pacing **(B)**, along with recorded electrograms. During intrinsic rhythm, late potentials lay dormant within the QRS or just post-QRS **(A)**, while during the sense protocol, late potentials are unmasked as shown in **B** and also the corresponding electrograms. Electrograms were recorded from the same area as shown by the high density grid in both maps. Highlighted areas in **yellow** indicate regions of local abnormal ventricular activity or late potentials.

**Figure 4:**
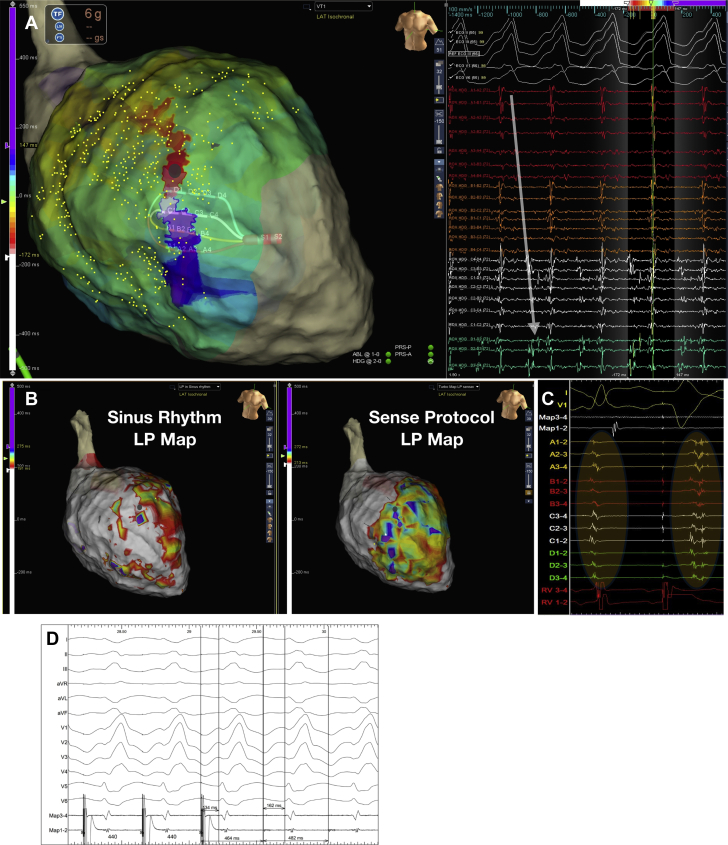
Relationship of Sense Protocol Functional Late Potentials to a Mapped VT Circuit **(A)** Activation mapping of ventricular tachycardia (VT) in a patient with previous anterior myocardial infarction as shown in Video 1. The high-density grid is placed on the critical isthmus, with mid-diastolic signals recorded on the right; it can be seen that the activation sequence goes from spline A to spline D, demarking the entrance and exit of the VT diastolic channel **(white arrow)**. **(B)** Late potentials (LP) color timing map during sinus rhythm **(left)** and sense protocol pacing **(right)** showing a greater region of LP during the sense protocol corresponding to the mapped diastolic pathway of VT. **(C)** Example of local LP delay and splitting of LP during sense protocol (second beat) along the mapped diastolic pathway of VT. **(D)** Entrainment from site close to pole D1 on the high-density grid **(gray dot)** shows concealed entrainment with a post-pacing interval of 18 ms, and a stimulus to QRS duration <30% VT cycle length, indicating entrainment at a VT exit site.

Figure 3 shows a comparison of LP and LAVA identified by the SP versus intrinsic rhythm in a sample patient. It can be seen that the SP identifies a larger region of LP that were not present during sinus rhythm substrate mapping (Figure 4). The median area of LP across the 30 patients during sinus rhythm was 6.4 mm^2^ (IQR 2 to 7 mm) during sinus rhythm mapping and 19.3 mm^2^ (IQR 7 to 25 mm) during SP pacing (p = 0.001); this represented a median of 9% of the total scar in sinus rhythm and 38% during SP.

### Functional areas of LP and LAVA relate to critical areas of the VT circuit

The functional unmasking of LP and LAVA was observed in 26 patients and showed good correlation to critical regions of the VT circuit (sites of best entrainment or pace map). The sensitivity and specificity value of SP to critical sites of ablation were 87% (95% CI: 85 to 89) and 96% (95% CI: 94 to 98), respectively, versus 78% (95% CI: 74 to 82) and 65% (95% CI: 63 to 67) for sinus rhythm mapping (Supplemental Figure 4).

Figure 4 presents an example of activation mapping of a VT circuit in a patient with ischemic cardiomyopathy and prior anterior myocardial infarction (Video 1). The HD grid was used to map the diastolic pathway. SP substrate mapping of this region shows better delineation of LP within the diastolic corridor through dynamic LP delay and unmasking of regions of functional slow conduction.

**Figure 5:**
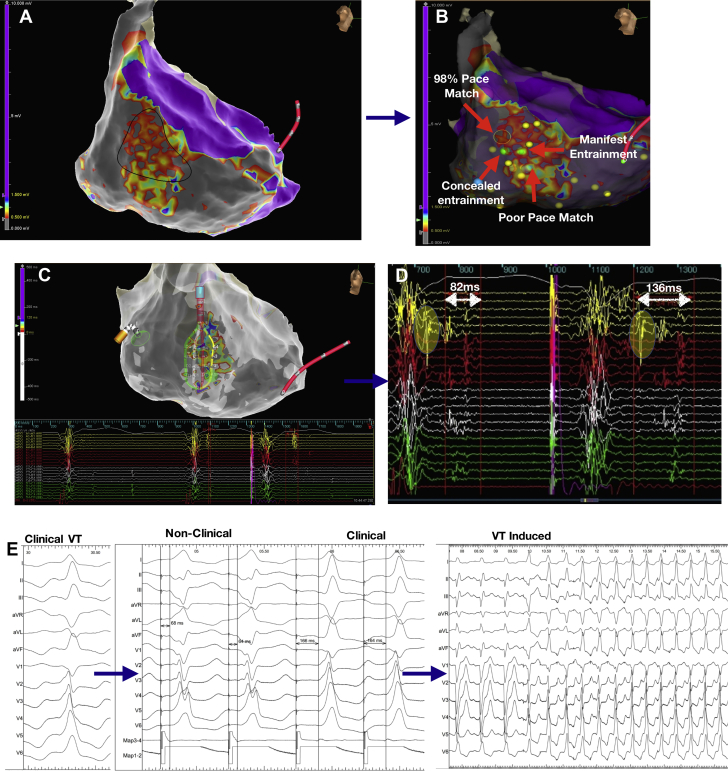
Sense Protocol Functional Late Potential Behavior in Critical Regions of the VT Circuit **(A)** Sense protocol voltage map with a highlighted region **(black shape)** of patchy low voltage within a region of dense scar. After substrate mapping, ventricular tachycardia (VT) was induced in this region (Video 2), and entrainment and pace mapping were performed. **(B)** Sites of entrainment and pace mapping, with yellow dots representing regions of late potentials (LP) marked manually. During substrate mapping, a sense protocol pacing map of LP was created. **(C)** The high-density grid with recorded signal over a region of LP. The timing of the LP is used to create an LP map, with color scale seen on the bar on the **left** and signal recorded shown **below**. **(D)** It can be seen that there is decrement in the LP region on sense protocol single extra pacing (second beat), compared with intrinsic rhythm (first beat), with timing markers post-QRS displayed. The region highlighted in yellow is local abnormal ventricular activity in intrinsic rhythm, which is delayed to the end of the QRS during sense protocol pacing. Video 3 shows the complex pattern of sense protocol LP conduction in this region with areas of block and complex wave front, which may explain the pace map and entrainment findings. **(E)** From left to right, clinical VT **(left panel)**, pace mapping at an isthmus region **(middle panel)** where there was initial short stimulus to QRS and poor QRS correlation to the clinical VT, followed by a longer stimulus to QRS and 98% pace match; the **right panel** shows pace match induction of VT in this region, indicating the catheter in a critical part of the circuit.

Figure 5 presents an example of a VT circuit mapped in a patient with anterior septal scar (Video 2); regions of good pace map and poor pace map are seen within a very small region within the scar. Within this region, there were LAVA and LP with decrements on SP pacing indicating functional conduction delay. The activation map of LP (Video 3) shows a complex activation pattern within this region of low voltage with lines of block in keeping with the pace map and entrainment findings. This matches the regions of pace mapping, in which a region of poor pace map near to a region of good pace map indicating a line of block or multiple exits was seen, followed by pace map induction of VT. Ablation to this region of LP rendered the VT noninducible.

## Discussion

This study uses a new high-density mapping catheter (Advisor HD Grid) and a unique pacing protocol (Barts SP) to better delineate VT substrate and identify new markers for substrate ablation. We made 2 main findings: 1) wave front–induced changes in scar voltage are not present when bipolar mapping using HD wave arrangement is used; and 2) functional LP and LAVA can be unmasked by the SP, enabling better delineation of critical regions for VT ablation that may not be visible during sinus rhythm. This unique delineation of functional substrate changes combined with activation or pace mapping needs further large-scale analysis, ideally in a randomized controlled trial.

### HD wave obviates the influence of directionality on scar characterization

Previous studies have suggested that pacing wave front influences scar delineation (15,16) and that the wave front influences scar morphology. Our study found no significant difference in scar area between sinus rhythm and SP mapping. This may be due to the HD wave algorithm and the ability to measure bipoles across splines, thus obviating the problem of “bipolar blindness” in which the wave front runs parallel to the splines, as recently reported (17). Our findings show that by having wave fronts mapped at right angles, activation and voltage can be mapped, thus avoiding the problems faced with directionality from previous studies using linear bipoles (16), and similar information may be obtained from other multipolar catheters if used in a variety of orientations; further investigation is required.

### Functional LP and local abnormal ventricular activity as a marker of dynamic slow conduction

Few studies have investigated wave front directionality and abnormal potentials (17, 18, 19). Brunckhorst et al. (19) reported significant variation in identification of abnormal potentials when pacing from the right atrium versus the right ventricle; however, linear bipolar catheters were used with larger electrode rings and larger inter-electrode spacing, thus potentially resulting in recording from nearby healthy tissue. In addition, several methods for LP identification have been previously used (3,10,20), making study comparisons difficult. LP mapped in sinus rhythm may also lack sensitivity and specificity (21,22) and may not be present in up to 29% of VT re-entry circuits and 46% of VT termination sites during sinus rhythm mapping (23,24).

Jackson et al. (10) and Porta-Sánchez et al. (9) have shown that DEEP may increase the specificity for identifying the VT diastolic pathway using an extrastimuli protocol at 600 ms during RV pacing. However, this approach has practical limitations because it requires mapping substrate over several drive trains to look for decrements, in a cohort of patients who are unwell with poor ventricular function, and this potentially increases procedure time. Similarly, evoked delayed potential mapping requires significant manual signal annotation (10). Our method allows for automated mapping of both sinus rhythm and SP simultaneously within existing mapping systems, without significant post-processing.

Our findings differ significantly from the DEEP mapping (9) in that the Barts SP identified a greater area of abnormal substrate for ablation compared with mapping in intrinsic rhythm, whereas DEEP mapping identifies an area two-thirds smaller than the mapped LP region in sinus rhythm. These differences may be explained by substrate and conduction velocity adaptation during drive train pacing, which may conceal more physiological abrupt conduction delays through action potential duration and sodium channel adaptation to repeated stimulation (25). In addition, because DEEP mapping is used to identify regions of functional delay rather than LP, it would naturally identify a smaller area. The majority of VT/ventricular fibrillation recorded on implantable cardioverter-defibrillator devices occurs due to single premature extrasystolic events (12,13), and thus studying the cardiac substrate in this maladapted physiological state as shown in our protocol may explain our findings of greater regions of LP and also better success rates versus sinus rhythm mapping alone.

### Role of combined SP mapping with entrainment/pace mapping to improve outcomes

Activation and entrainment mapping of VT remain a challenge in patients in whom the VT is poorly tolerated, not inducible, or nonsustained. In addition, VT induction and mapping before substrate ablation have been shown to prolong procedure time with poorer outcomes (26,27). Several substrate-guided approaches have been devised, including linear ablation (2), LP mapping (3), and LAVA elimination (4). However, real-world outcomes from VT ablation remain as poor as 40% to 60% (7,28) regardless of ablation strategy. This highlights the limitations of present strategies in defining critical regions for ablation.

The Barts SP, which involves single extrastimuli at an interval 20 ms above ERP from the right ventricle, enables conduction delay to be invoked from the patients’ intrinsic steady state of conduction, thus looking specifically for maladaptation in regions of diseased tissue and slow conduction. Our study showed the ability of the SP to unmask dormant LP or LAVA (Figures 3, 4, and 5). In 80% of cases, these regions lay within 10 mm of critical sites for ablation, which is consistent with previous studies (23); however, we were able to see these sites in 87% of patients during sense mapping, and this potentially provides a new and practical method to map LP. The combination of SP substrate mapping with entrainment/pace mapping may, therefore, explain our symptomatic therapy–free rate of 90%. Further large, randomized comparator studies are required to corroborate this theory. Although focal targeting of critical regions may be desired from a practical perspective to reduce procedure time, careful and systematic analysis of the substrate and ablation at all potential regions of abnormal VT substrate, which may form substrates for alternative VT circuits in the future, is required.

### Study limitations

Accepted voltage cutoffs for scar threshold were applied based on published data; however, debate exists as to what the correct optimal voltage cutoffs should be. This is a nonrandomized single cohort of 30 patients; a randomized study is needed to further confirm its clinical value and compare outcomes. For clinical simplicity and patient safety, we only performed single extra pacing in the RV apex, and whether our findings would be reproducible and/or perform better with pacing from other sites needs further investigation. The paper is only descriptive and does not directly make comparisons with other multipolar catheters or substrate ablation strategies. We did not systematically compare RV drive train or S1/S2 pacing versus single extra pacing (Supplement 5).

## Conclusions

Our novel pacing protocol (Barts SP) improved identification of functional abnormal substrate, which was able to improve delineation of critical areas for VT ablation. Larger multicenter randomized studies are required to assess the success of this method of ablation versus conventional strategies.

Perspectives **COMPETENCY IN MEDICAL KNOWLEDGE:** LP, which are a marker of slow conduction within ventricular scar and may contribute to the VT isthmus, are dynamic in nature. This information may be useful in guiding substrate-based catheter ablation strategies.

**TRANSLATIONAL OUTLOOK:** Larger multicenter randomized studies are required to evaluate the success of this method of ablation against conventional strategies. Investigation of the effect of different pacing protocols on LP is required.

## Author Disclosures

This work was supported by University College London Hospitals Biomedicine National Institute for Health Research. Dr. Srinivasan was supported by a British Heart Foundation Clinical Research Training Fellowship (FS/14/9/30407). Dr. Lambiase was supported by the Medical Research Council (G0901819), Barts BRC, and the Stephen Lyness Research Fund. Drs. Srinivasan, Chow, Lowe, Schilling, and Lambiase have received speaker fees from Abbott in the last 10 years. Dr. Lambiase has received research grants from Boston Scientific and Abbott. All other authors have reported that they have no relationships relevant to the contents of this paper to disclose.

## Appendix

Supplemental Data

Supplemental Video 1 VT Activation Map

Supplemental Video 2 VT Activation Map

Supplemental Video 3 Sense Protocol Propagation Map Over VT Critical Region

## References

[1] Stevenson W.G., Friedman P.L., Sager P.T. (1997). Exploring postinfarction reentrant ventricular tachycardia with entrainment mapping. J Am Coll Cardiol.

[2] Marchlinski F.E., Callans D.J., Gottlieb C.D., Zado E. (2000). Linear ablation lesions for control of unmappable ventricular tachycardia in patients with ischemic and nonischemic cardiomyopathy. Circulation.

[3] Arenal A., Glez-Torrecilla E., Ortiz M. (2003). Ablation of electrograms with an isolated, delayed component as treatment of unmappable monomorphic ventricular tachycardias in patients with structural heart disease. J Am Coll Cardiol.

[4] Sacher F., Lim H.S., Derval N. (2015). Substrate mapping and ablation for ventricular tachycardia: the LAVA approach. J Cardiovasc Electrophysiol.

[5] Vergara P., Trevisi N., Ricco A. (2012). Late potentials abolition as an additional technique for reduction of arrhythmia recurrence in scar related ventricular tachycardia ablation. J Cardiovasc Electrophysiol.

[6] Kumar S., Baldinger S.H., Romero J. (2016). Substrate-based ablation versus ablation guided by activation and entrainment mapping for ventricular tachycardia: a systematic review and meta-analysis. J Cardiovasc Electrophysiol.

[7] Kuck K.H., Schaumann A., Eckardt L. (2010). Catheter ablation of stable ventricular tachycardia before defibrillator implantation in patients with coronary heart disease (VTACH): a multicentre randomised controlled trial. Lancet.

[8] Srinivasan N.T., Orini M., Providencia R. (2019). Prolonged action potential duration and dynamic transmural action potential duration heterogeneity underlie vulnerability to ventricular tachycardia in patients undergoing ventricular tachycardia ablation. Europace.

[9] Porta-Sánchez A., Jackson N., Lukac P. (2018). Multicenter study of ischemic ventricular tachycardia ablation with decrement-evoked potential (DEEP) mapping with extra stimulus. J Am Coll Cardiol EP.

[10] Jackson N., Gizurarson S., Viswanathan K. (2015). Decrement evoked potential mapping: basis of a mechanistic strategy for ventricular tachycardia ablation. Circ Arrhythm Electrophysiol.

[11] De Riva M., Naruse Y., Ebert M. (2018). Targeting the hidden substrate unmasked by right ventricular extrastimulation improves ventricular tachycardia ablation outcome after myocardial infarction. J Am Coll Cardiol EP.

[12] Roelke M., Garan H., McGovern B.A., Ruskin J.N. (1994). Analysis of the initiation of spontaneous monomorphic ventricular tachycardia by stored intracardiac electrograms. J Am Coll Cardiol.

[13] Saeed M., Link M.S., Mahapatra S. (2000). Analysis of intracardiac electrograms showing monomorphic ventricular tachycardia in patients with implantable cardioverter-defibrillators. Am J Cardiol.

[14] Bogun F., Good E., Reich S. (2006). Isolated potentials during sinus rhythm and pace-mapping within scars as guides for ablation of post-infarction ventricular tachycardia. J Am Coll Cardiol.

[15] Tung R., Bradfield J., Buch E. (2015). Influence of ventricular pacing wavefronts on myocardial scar detection during electroanatomic voltage mapping. J Am Coll Cardiol.

[16] Tung R., Josephson M.E., Bradfield J.S., Shivkumar K. (2016). Directional influences of ventricular activation on myocardial scar characterization. Circ Arrhythm Electrophysiol.

[17] Takigawa M., Relan J., Martin R. (2018). Effect of bipolar electrode orientation on local electrogram properties. Heart Rhythm.

[18] Brunckhorst C.B., Delacretaz E., Soejima K. (2004). Ventricular mapping during atrial and right ventricular pacing: relation of electrogram parameters to ventricular tachycardia reentry circuits after myocardial infarction. J Interv Card Electrophysiol.

[19] Brunckhorst C.B., Stevenson W.G., Jackman W.M. (2002). Ventricular mapping during atrial and ventricular pacing. Relationship of multipotential electrograms to ventricular tachycardia reentry circuits after myocardial infarction. Eur Heart J.

[20] Jaïs P., Maury P., Khairy P. (2012). Elimination of local abnormal ventricular activities: a new end point for substrate modification in patients with scar-related ventricular tachycardia. Circulation.

[21] Hsia H.H., Lin D., Sauer W.H., Callans D.J., Marchlinski F.E. (2009). Relationship of late potentials to the ventricular tachycardia circuit defined by entrainment. J Interv Card Electrophysiol.

[22] Irie T., Yu R., Bradfield J.S. (2015). Relationship between sinus rhythm late activation zones and critical sites for scar-related ventricular tachycardia. Circ Arrhythm Electrophysiol.

[23] Harada T., Stevenson W.G., Kocovic Dz, Friedman Pl (1997). Catheter ablation of ventricular tachycardia after myocardial infarction: relation of endocardial sinus rhythm late potentials to the reentry circuit. J Am Coll Cardiol.

[24] Anter E., Kleber A.G., Rottmann M. (2018). Infarct-related ventricular tachycardia: redefining the electrophysiological substrate of the isthmus during sinus rhythm. J Am Coll Cardiol EP.

[25] Franz M.R. (2003). The electrical restitution curve revisited: steep or flat slope—which is better?. J Cardiovasc Electrophysiol.

[26] Fernández-Armenta J., Penela D., Acosta J. (2016). Substrate modification or ventricular tachycardia induction, mapping, and ablation as the first step? A randomized study. Heart Rhythm.

[27] Di Biase L., Burkhardt J.D., Lakkireddy D. (2015). Ablation of stable VTs versus substrate Ablation in ischemic cardiomyopathy: the VISTA Randomized Multicenter Trial. J Am Coll Cardiol.

[28] Breitenstein A., Sawhney V., Providencia R. (2019). Ventricular tachycardia ablation in structural heart disease: impact of ablation strategy and non-inducibility as an end-point on long term outcome. Int J Cardiol.

