# Supplementary material for: Multicenter Study of Dynamic High-Density Functional Substrate Mapping Improves Identification of Substrate Targets for Ischemic Ventricular Tachycardia Ablation
Source: JACC Clin Electrophysiol. 2020 Dec;6(14):1783–93. doi: 10.1016/j.jacep.2020.06.037 (PMC7769061; doi:10.1016/j.jacep.2020.06.037)
Supplement: Supplemental Data [file mmc1.docx]

# Sensitivity Specificity & Predictive Value Results

Multiple logistic regression was performed for all late potentials (LP) that were within critical regions for ablation and outside critical regions for ablation during sense protocol pacing and during sinus rhythm. This was conducted using the glm function in the R statistic package in a stepwise approach. Sex, age, ejection fraction, hypertension, prior stroke, chronic kidney disease, diabetes and etiology of VT were incorporated into the multiple regression model, in a stepwise manner. None of these factors improved the model or significantly correlated with the presence of LP within critical regions, therefore LP within and outside critical regions for ablation were incorporated into their respective models for sinus rhythm and sense protocol mapping. This model was then used to develop ROC curves, the data for which are shown below (sense protocol = blue, sinus rhythm = green).

It can be seen the sense protocol mapping had a better AUC, sensitivity, specificity and true positive percentage (TPP) and false positive percentage (FPP) than sinus rhythm mapping.

Below is proportion if late potentials located within critical areas for ablation in the sense protocol VS sinus rhythm.

| Patient No | Percentage Sense Protocol | Percentage Sinus Rhythm |
| --- | --- | --- |
| 1 | 58 | 8 |
| 2 | 85 | 0 |
| 3 | 82 | 13 |
| 4 | 78 | 3 |
| 5 | 74 | 21 |
| 6 | 88 | 27 |
| 7 | 95 | 0 |
| 8 | 98 | 23 |
| 9 | 88 | 3 |
| 10 | 77 | 33 |
| 11 | 87 | 40 |
| 12 | 79 | 32 |
| 13 | 83 | 50 |
| 14 | 79 | 62 |
| 15 | 75 | 40 |
| 16 | 85 | 59 |
| 17 | 75 | 33 |
| 18 | 76 | 70 |
| 19 | 86 | 52 |
| 20 | 85 | 6 |
| 21 | 91 | 64 |
| 22 | 91 | 63 |
| 23 | 85 | 43 |
| 24 | 91 | 32 |
| 25 | 87 | 45 |
| 26 | 86 | 75 |
| 27 | 58 | 55 |
| 28 | 93 | 45 |
| 29 | 91 | 32 |
| 30 | 70 | 31 |
| Percentage Average | 82 | 35 |

**Effects Of Steady State RV Pacing**

We did not formally assess the effect of steady state RV pacing vs single extra (sense protocol) vs sinus rhythm mapping on late potentials and LAVA. This was because we did not want to spend extra time performing three substrate maps in a group of patients who are unwell. The point of the study was to compare our method to standard sinus rhythm mapping, and this was performed via the turbo mapping facility of the mapping system therefore mapping times are similar to sinus rhythm substrate mapping. This is one of the advantages of the sense protocol.

We also highlight some of the difficulties and issues with RV steady state pacing with example below. Not only can there be late potential decrement and block during RV pacing, but sometimes late potentials may not be seen during RV pacing and be seen during sinus rhythm or single extra (sense protocol pacing). VT is often initiated by single extra beats in device tracings and we believe this method is a good representation of physiological situations where VT occurs in patients.

| Sex | Age | EF | HTN | STROKE | CKD | Diabetes | Betablocker | Amiodarone | 2 AAD | Etiology | Re-do |
| --- | --- | --- | --- | --- | --- | --- | --- | --- | --- | --- | --- |
| M | 61 | 28 | y | n | y | y | y | y | y | ihd | n |
| F | 53 | 34 | n | n | y | y | y | n | n | ihd/dcm | n |
| M | 84 | 20 | y | y | y | y | y | y | y | ihd | y |
| M | 66 | 20 | y | n | y | y | y | n | n | ihd | y |
| M | 69 | 20 | y | n | n | n | y | n | n | ihd | n |
| M | 66 | 22 | y | n | n | n | y | n | n | ihd | n |
| M | 61 | 32 | y | n | n | y | y | y | y | ihd | n |
| M | 74 | 20 | y | n | n | y | y | y | y | ihd | n |
| M | 62 | 60 | y | n | y | y | y | n | n | ihd | n |
| M | 55 | 24 | n | n | n | n | y | n | n | ihd | n |
| M | 61 | 20 | n | n | n | y | y | y | y | ihd | n |
| M | 77 | 12 | y | b | y | y | y | y | y | ihd | y |
| F | 81 | 36 | y | n | n | n | y | n | n | ihd/dcm | n |
| M | 51 | 38 | y | y | n | y | y | y | y | ihd | n |
| M | 76 | 15 | y | n | n | n | n | y | n | ihd | n |
| M | 64 | 23 | y | y | n | y | y | n | n | ihd | n |
| M | 72 | 10 | y | n | n | n | y | y | y | ihd | n |
| M | 72 | 22 | y | n | n | y | y | y | y | ihd | n |
| M | 66 | 18 | y | n | n | n | y | y | y | ihd | n |
| M | 71 | 27 | t | n | n | n | y | y | y | ihd | n |
| M | 71 | 33 | y | n | n | y | y | y | y | ihd | n |
| M | 68 | 20 | y | n | n | n | y | n | n | ihd | n |
| M | 48 | 17 | y | n | n | n | y | y | y | ihd | n |
| F | 68 | 25 | y | n | n | y | y | n | n | ihd | n |
| M | 72 | 28 | y | n | y | y | y | n | n | ihd | n |
| M | 62 | 33 | y | n | y | n | y | n | n | ihd | n |
| M | 69 | 26 | n | n | n | n | y | y | y | ihd | n |
| M | 80 | 27 | n | y | n | n | y | n | n | ihd | n |
| M | 78 | 14 | y | n | n | n | y | y | y | ihd | n |
| M | 57 | 20 | y | n | n | n | y | y | y | ihd | n |

Supplementary Table 1. Patient Demographics

**Supplementary Table 2**

**VT Burden Pre and Post Ablation For Each Patient**

**Total number of VT events in the 6 month period prior to ablation and in the follow up period**

| VT 6months Pre | VT POST | Follow up Months |
| --- | --- | --- |
| 12 | 0 | 21 |
| 98 | 6 | 20 |
| 295 | 35 | 9 |
| 20 | 32 | 13 |
| 12 | 0 | 15 |
| 106 | 1 | 16 |
| 42 | 1 | 16 |
| 67 | 0 | 15 |
| 929 | 6 | 15 |
| 24 | 0 | 15 |
| 44 | 0 | 14 |
| 40 | 35 | 9 |
| 26 | 0 | 13 |
| 256 | 15 | 13 |
| 28 | 0 | 13 |
| 43 | 0 | 13 |
| 32 | 2 | 13 |
| 46 | 0 | 11 |
| 45 | 1 | 9 |
| 36 | 4 | 9 |
| 9 | 0 | 8 |
| 12 | 0 | 9 |
| 27 | 0 | 7 |
| 15 | 0 | 9 |
| 6 | 0 | 10 |
| 12 | 0 | 11 |
| 10 | 0 | 7 |
| 5 | 0 | 7 |
| 12 | 0 | 8 |
| 375 | 0 | 7 |

| Mean | 89.4 | 4.6 | 12 |
| --- | --- | --- | --- |

| Shock 6 months Pre | Shocks Post | Follow up Months |
| --- | --- | --- |
| 0 | 0 | 21 |
| 0 | 0 | 20 |
| 16 | 2 | 9 |
| 1 | 2 | 13 |
| 3 | 0 | 15 |
| 0 | 0 | 16 |
| 22 | 0 | 16 |
| 0 | 0 | 15 |
| 0 | 0 | 15 |
| 2 | 0 | 15 |
| 30 | 0 | 14 |
| 9 | 4 | 9 |
| 1 | 0 | 13 |
| 3 | 0 | 13 |
| 2 | 0 | 13 |
| 2 | 0 | 13 |
| 6 | 0 | 13 |
| 4 | 0 | 11 |
| 6 | 0 | 9 |
| 7 | 0 | 9 |
| 0 | 0 | 8 |
| 2 | 0 | 9 |
| 1 | 0 | 7 |
| 2 | 0 | 9 |
| 1 | 0 | 10 |
| 0 | 0 | 11 |
| 4 | 0 | 7 |
| 1 | 0 | 7 |
| 3 | 0 | 8 |
| 5 | 0 | 7 |

| Mean | 4.4 | 0.27 | 12 |
| --- | --- | --- | --- |

**Supplemental Table 3**

**ICD Shocks Pre Post Ablation For Each Patient**

**Total number ICD Shocks in the 6 month period prior to**

**ablation and in the follow up period**
